# Supplementary material for: P-TRAP: a Panicle Trait Phenotyping tool
Source: BMC Plant Biol. 2013 Aug 29;13:122. doi: 10.1186/1471-2229-13-122 (PMC3848748; doi:10.1186/1471-2229-13-122)
Supplement: Additional file 7 — P-TRAP source code. The Netbeans project that contains the source code of the application. [file 1471-2229-13-122-S7.zip › javadoc.zip!/javadoc/org-netbeans-modules-riceoptions/index.html]

Rice Options


<noscript>
<div>JavaScript is disabled on your browser.</div>
</noscript>
<h2>Frame Alert</h2>
<p>This document is designed to be viewed using the frames feature. If you see this message, you are using a non-frame-capable web client. Link to <a href="org/netbeans/modules/riceoptions/package-summary.html">Non-frame version</a>.</p>
